# Assessment of an electronic patient record system on discharge prescribing errors in a Tertiary University Hospital

**DOI:** 10.1186/s12911-021-01551-5

**Published:** 2021-06-21

**Authors:** Michael Patrick O’Shea, Cormac Kennedy, Eileen Relihan, Kieran Harkin, Martina Hennessy, Michael Barry

**Affiliations:** 1 Department of Pharmacology, Saint James Hospital, Dublin, Ireland; 2 Dublin Southeast Network Academic Track Internship, Dublin, Ireland; 3 School of Medicine, Trinity College Dublin, Dublin, Ireland; 4 Inchicore Family Doctors, Dublin, Ireland

**Keywords:** Discharge, Prescribing, Transition, E-health, Medical education

## Abstract

**Background:**

Prescribing error represent a significant source of preventable harm to patients. Prescribing errors at discharge, including omission of pre-admission medications (PAM), are particularly harmful as they frequently propagate following discharge. This study assesses the impact of an educational intervention and introduction of an electronic patient record (EPR) in the same centre on omission of PAM at discharge using a pragmatic design. A survey of newly qualified doctors is used to contextualise findings.

**Methods:**

Discharge prescriptions and discharge summaries were reviewed at discharge, and compared to admission medicine lists, using a paper-based chart system. Discrepancies were noted, using Health Information and Quality Authority guidelines for discharge prescribing. An educational intervention was conducted. Further review of discharge prescriptions and discharge summaries took place. Following introduction of an EPR, review of discharge summaries and discharge prescriptions was repeated. A survey was administered to recently qualified doctors (interns), and analysed using descriptive statistics and thematic analysis.

**Results:**

Omission of PAM as prescribed or discontinued items at discharge occurs frequently. An educational intervention did not significantly change prescribing error rates (U = 1255.5, *p* = 0.206). EPR introduction did significantly reduce omission of PAM on discharge prescribing (U = 694, *p* < 0.001), however there was also a reduction in the rate of deliberate discontinuation of PAM at discharge (U = 1237.5, *p* = 0.007). Survey results demonstrated that multiple sources are required to develop a discharge prescription. Time pressure, access to documentation and lack of admission medicine reconciliation are frequently cited causes of discharge prescribing error.

**Conclusion:**

This study verified passive educational interventions alone do not improve discharge prescribing. Introduction of EPR improved discharge prescribing, but negatively impacted deliberate discontinuation of PAM at discharge. This is attributable to reduced access to key sources of information used in formulating discharge prescriptions, and separation of the discontinuation function from the prescribing function on the EPR discharge application.

**Supplementary Information:**

The online version contains supplementary material available at 10.1186/s12911-021-01551-5.

## Background

A prescribing error occurs when, as a result of a prescribing decision or prescription writing process, there is an unintentional significant reduction in the probability of a treatment being timely and effective, or increase in the risk of harm [1]. Prescribing errors, including omission of pre-admission medications (PAM) represent a significant source of preventable harm to patients [2–5]. While much focus has been on defining preventable prescribing errors during inpatient episodes, a significant number of errors occur at transition from secondary to primary care [6–9]. These events are very likely to propagate in the community following discharge [6]. Hospital-based prescriptions account for a large proportion of community prescribing in Ireland. These prescriptions often do not easily translate into a clear discharge medication regimen as many omitted medicines, which were held during admission for acute care purposes are not appropriately restarted on discharge [10]. The subsequent lack of clarity is a source of frustration to General Practitioners (GPs) and Community Pharmacists [11, 12]. Further, errors such as unintended discontinuation of a medication, have been clearly and directly linked to inadequate documentation at discharge [10].

The World Health Organisation has identified transitions of care as a Key Action Area in the Global Patient Safety Challenge of reducing iatrogenic medication harm by 50% by 2022 [13]. Actions which may address medication safety at transition of care include pharmacist-led Medicine Reconciliation (MR) at admission and discharge, patient held medication lists, information technologies such as electronic patient records (EPR), and educational interventions.

A number of these interventions have demonstrated promising results when implemented in practice. MR at discharge is very effective in addressing discharge prescribing errors and in improving communication between Hospital Physicians and General Practitioners [6, 14–17]. Data on the cost effectiveness of MR at discharge is limited, and the need for additional pharmacy staff may limit implementation in certain health systems [17–19]. Patient held medication lists encourage patient involvement in care, as part of the chronic care model. Patients who understand their medications at discharge are less likely to be readmitted [20]. Data on the impact of didactic educational interventions for prescribers is limited to studies which show improvements in standardised assessments, with limited impact on observed prescribing error rates [21–24]. Information technology has largely had a positive impact on discharge prescribing error, however results are not consistent across all studies, and there is substantial variance in the electronic systems studied [25–27].

### Aims

This study aims to assess an educational intervention and an electronic intervention to improve discharge prescribing in the same institution, in a planned, pragmatic observational manner. It assesses the impact these interventions have on discharge prescribing practices in Saint James’s Hospital (SJH), Ireland’s largest Tertiary University Teaching Hospital. A passive educational intervention is used as this method is the conventional mechanism used to address issues relating to areas of care managed by trainee doctors (interns). This paper aims to rationalise findings using attitudes and opinions of interns on the topic of discharge prescribing safety.

## Methods

### Study design

This prospective cohort study was a pragmatic study to identify prescribing errors which were noticed by local GPs and brought to the attention of the SJH Medication Safety Officer. A timeline for introduction of EPR to all inpatient services was available at that point, however implementation was not imminent. A review of current practices (see Data Collection), followed by an educational intervention, was planned, with a view to immediately addressing patient safety concerns. The educational intervention relied on standard hospital medication safety practices to ensure generalizability. This allowed comparison of an educational program and introduction of EPR in the same setting. Following the educational intervention, a further review of current practices was conducted. After EPR was introduced, a final review of current practice was conducted. The design was pragmatic so that normal hospital processes and the patients were not affected.

A survey was conducted to determine attitudes of interns (junior doctors in their first year of training) towards prescribing errors at point of discharge. Interns were selected as a focus for this study as they are most frequently tasked with preparing discharge prescriptions and discharge summaries. This survey was piloted for ease of use among final year medical students and adjusted for ease of use. This survey consisted of quantitative and qualitative Likert-style questions. It was administered at scheduled weekly teaching session prior to any educational intervention on discharge prescribing errors. Institutional Approval was obtained from the Saint James’s Hospital Research and Innovation Committee.

### Setting

SJH is Ireland’s largest Tertiary University Teaching Hospital (with approximately 1000 inpatient beds). SJH employs 52 interns per year. Interns in Ireland complete a 1 year training program before progressing to subspecialist roles. SJH provides all major services with the exception of paediatrics and obstetrics. In SJH in 2017, there were no electronic patient medical records, and discharge prescribing was completed by hand-written prescription. Hospital notes were documented in paper charts. Medications were prescribed on paper. Discharge summaries were typed electronically. In SJH, MR takes place at admission by ward pharmacists. There is no pharmacist-led reconciliation at discharge.

In October 2018, SJH adopted an EPR system for all inpatient services, which includes inpatient and discharge prescribing. The EPR introduced in Saint James’s Hospital contained discharge section with 2 action tabs for medication; “Medication Reconciliation” and “Medication Changes and Comments”, in addition to other action tabs necessary for safe discharge.

- The Medicine Reconciliation contains a computer-generated Discharge Medicine Reconciliation tab to be completed by the physician. This lists all inpatient medications, as well as all Pre-Admission Medications (PAM) identified on Admission Medicine Reconciliation, and which is used to generate a discharge prescription. As part of this process, prescribers can include notes about items which are prescribed. It is not possible to discontinue medications in this tab. This was a notable limitation of EPR discharge prescribing.
- The Medication Changes and Comments section is a separate tab which consists of three text boxes for the following information:
- PAM that were Stopped/Changed and Why
- New Medications Started this Admission and Why
- Additional Medicines Comments

### Data collection

Discharge summaries and discharge prescriptions were reviewed at point of discharge on wards and in the hospital Discharge Lounge using convenience sampling. Inclusion criteria included:

- a completed MR on admission,
- completed discharge prescription and discharge summary, and
- presence of ≥ 1 Pre-Admission Medication (PAM).

To quantify the rate at which PAM are omitted or discontinued without explanation, admission MR was compared to discharge prescriptions and discharge summaries, and discrepancies noted (MOS, CK).

Health Information and Quality Authority guidelines on Discharge Summary Information were used to define acceptable practice [28]. PAM were classified as “Prescribed”, “Omitted” (not prescribed, and not listed as a discontinued medication on either the discharge prescription or discharge summary), “Discontinued with Explanation” and “Discontinued without Explanation”. Composite primary outcome was proportion of PAM which were Prescribed and Discontinued with Explanation expressed as a proportion of all PAM (Proportion Correctly Prescribed on Discharge—PCPD). PAM which were omitted or discontinued without explanation, were considered incorrectly prescribed at discharge, and expressed as a proportion of PAM are referred to as Proportion Incorrectly Prescribed on Discharge (PIPD).

### Interventions

A review of discharge prescriptions and discharge summaries was initially conducted in August and September 2017 (Pre-Intervention Review), and data from this cohort is used as a control. An awareness campaign targeting first year junior doctors (interns) was subsequently implemented in SJH, as interns are typically tasked with discharging patients.

This educational intervention consisted of:

- A mandatory multidisciplinary teaching session led by a GP, a pharmacist, the medication safety officer and the Department of Clinical Pharmacology and Therapeutics.
- Posters promoting good discharge prescribing practice placed in opportune locations throughout the hospital, including the Discharge Lounge and Acute Medical Assessment Unit.
- An information bulletin circulated via junior doctor social media.

The objective of the teaching session was to illustrate the HIQA National Standards for Patient Discharge Summary Information, discuss current practice, and outline an appropriate processes for preparing a discharge prescription and discharge summary. Emphasis was placed on improved continuity of care through comprehensive handover regarding medication. The latter two interventions were conducted after the didactic and were intended to reinforce the concepts discussed. A subsequent review of discharge prescriptions and discharge summaries was conducted after this campaign (Post-Education Review).

**Fig. 1:**
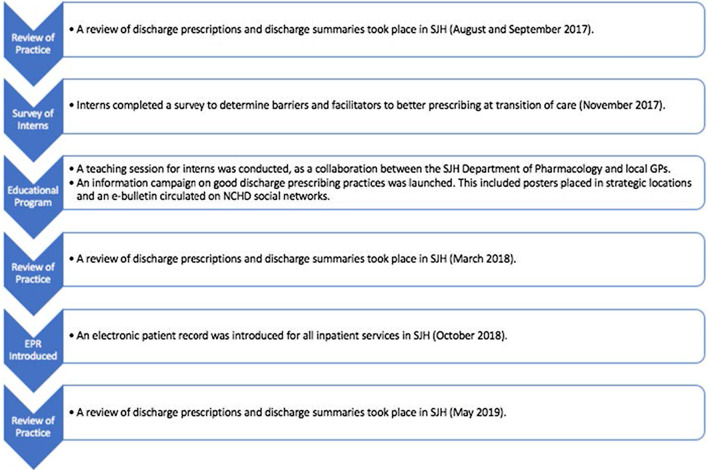
Timeline of events

An EPR was introduced for all inpatient services. A third review of discharge prescriptions and discharge summaries was conducted (Post-EPR Review). This was conducted 6 months after the introduction of EPR to restrict the impact of learning to a new system on outcomes. See Fig. 1 for a full timeline of events (Microsoft Word v16.36).

### Statistical analysis

Chi-squared analysis was used to assess for difference in the number of medications which were prescribed, omitted, discontinued with explanation and discontinued without explanation among the three reviews. Levene’s test was used to assess for equality of variance in individual and composite outcomes. Mann–Whitney-U test was conducted to assess for difference in the proportion of PAM which were correctly communicated at discharge. Bivariate Correlation using Pearson’s Correlation Coefficient was used to assess relationship between possible confounding variables (number of PAM and length of stay) and a number of outcomes (Proportion of PAM which were omitted, Proportion of PAM which were discontinued without explanation, Proportion of PAM which were Documented Correctly on Discharge Prescription). Where normal distribution was identified, data was considered unpaired and two-tailed analysis was conducted. Data was analysed, and figures generated using SPSS Statistics v26 (IBM corp, Armonk, NY, USA).

With regard to survey data, quantitative and qualitative components were analysed separately. Quantitative Likert-style questions were analysed using descriptive statistics. Qualitative components were analysed using thematic analysis. Data was analysed using Microsoft Excel v16.36. Tables were generated using Microsoft Word v16.36.

This project was approved by the Department of Research and Innovation in Saint James’s Hospital (ref 5273). Appropriate approval was obtained for surveying hospital staff per Saint James’s Hospital Protocol. Consent to participate in the survey and teaching session was obtained from participants. Written consent was obtained from participants who completed a survey. All methods were performed in accordance with local and national guideline and regulations. European Union General Data Protection Regulation was strictly adhered to, and all researchers involved in data collection and analysis completed training in GDPR compliance and data protection principals.

## Results

### Changes in prescribing practices with education and introduction of electronic patient records

54 prescriptions were reviewed each at Pre-Intervention Review, Post-Education Review and Post-EPR review. In total, *n* = 1218 PAM were reviewed. See Table 1 for breakdown of results.

**Table 1:** Outcome of all pre-admission medications recorded on discharge prescriptions and discharge summaries

Audit (n = number of prescriptions reviewed)	Total pre-admission medications	Prescribed	Omitted	Discontinued with explanation	Discontinued without explanation
Pre-intervention (n = 54)	436	299 (68.58%)	116 (26.61%)	10 (2.29%)	11 (2.52%)
Post-education (n = 54)	443	323 (72.91%)	85 (19.19%)	24 (5.42%)	11 (2.48%)
Post-EPR (n = 54)	339	312 (92.04%)	26 (7.67%)	1 (0.29%)	0 (0%)

#### Impact of an educational intervention

70.87% of PAM were correctly prescribed (prescribed or discontinued with explanation) on Pre-Intervention Review, compared to 78.33% following educational intervention, and this difference was not statistically significant (U = 1255.5, *p* = 0.206). Across all 4 possible outcomes (prescribed, omitted, discontinued with and without explanation) for a pre-admission medication, there was no significant change compared to expected values following the Educational Intervention (χ^2^ = 6.4184, *p* = 0.093). The percentage of medications which were discontinued with explanation relative to all discontinued medications was 47.6% (11/21) on Pre-Intervention Review, compared to 68.6% (24/35) on Post-Education Review, however, this was not statistically significant (U = 1378, *p* = 0.468).

#### Impact of introduction of EPR

**Fig. 2:**
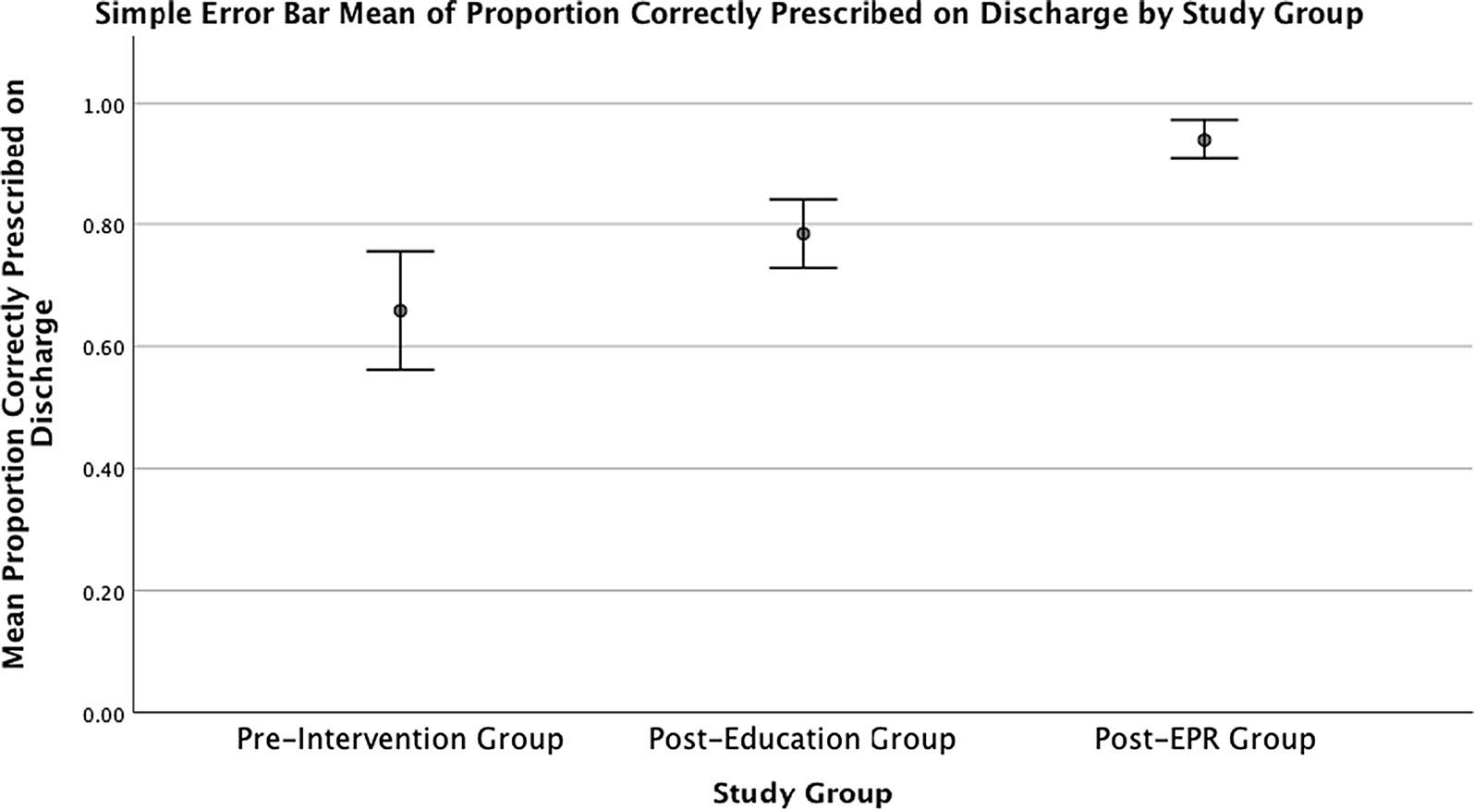
Simple Error Bar Chart of proportion of pre-admission medications which were correctly documented on discharge

The PCPD following introduction of EPR was 92.33%, and this was significantly greater than Pre-Intervention (U = 694, *p* < 0.001) and Post-Education Review (U = 731, *p* < 0.001) (see Fig. 2). Across all 4 possible outcomes (prescribed, omitted, discontinued with and without explanation) for a pre-admission medication, there was a significant change following the introduction of EPR, compared to pre-intervention values (χ^2^ = 25.65 > χ^c^ = 7.82, *p* < 0.001). However, of concern, only one of the PAM were discontinued with explanation on Post-EPR review, and this was significantly less than on Pre-Intervention Review (U = 1237.5, *p* = 0.007).

#### Assessment for confounding variables

There was a relative decrease in total number of PAM in the Post-EPR group. To determine the impact this had on the study results overall, data from all three groups was analysed. There was no significant association between number of PAM and PCPD (r = 0.008, *p* = 0.916). There was no significant association between number of PAM and proportion of PAM which were omitted (r = − 0.23, *p* = 0.771), or proportion of PAM which were discontinued without explanation (r = 0.064, *p* = 0.421).

Duration of Stay in Hospital was assessed in terms of impact on outcomes for all three groups. There was no significant association between Duration of Stay and PCPD (r = − 0.02, *p* = 0.804, n = 162), proportion of PAM which were omitted (r = 0.02, *p* = 0.801), or the proportion of PAM which were discontinued without explanation (r = − 0.046, *p* = 0.561).

### Attitudes of interns towards prescribing changes

A written survey was conducted at an Intern Teaching Session on the 9th of January 2018. All interns at this time were using a paper-based chart system to complete typed discharge summaries and handwritten discharge summaries. Thirty-three interns attended this session (of forty-two interns who were available to attend), of which n = 31 completed or partially completed the survey. Quantitative and Qualitative results are outlined below.

#### Qualitative survey questions

Interns were asked to suggest why they thought a number of different prescribing errors occurred on discharge. Space was provided to list multiple items (see Table 2). Omission of medications at discharge was attributed to omission of medications on admission, unclear documentation of medication changes and poor handwriting. Errors relating to frequency, formulation and dose were attributed to poor handwriting, lack of familiarity with certain medications, and time pressure. Prescription of a medication that was intentionally stopped during inpatient stay was most frequently attributed to inadequate inpatient documentation of the discontinuation, and lack of familiarity with patient notes.

**Table 2:** Reasons identified by interns as causes of different types of discharge prescribing errors

Omission of medications (n = 21)	Errors relating to frequency, dose or formulary (n = 19)
Omission of medications on admission (7) Unclear documentation of medication changes (4) Poor handwriting (3) Multiple medication prescription charts used (3) Time pressure (3) Distractions (i.e. being paged) while discharging (2) Unfamiliar with patient (1) Polypharmacy (1)	Poor handwriting (6) Lack of familiarity with certain medications (4) Time pressure (4) Lack of familiarity with patients (2) Failure to check guidelines/formularies (2) Difficulty accessing admission medication list during a prolonged admission (2) Human error (2) Unclear documentation of medication changes (1) Inexperience (1)

Communication error (n = 9)	Prescription of a medication that was intentionally stopped during inpatient stay (n = 15)
Time pressure (3) Unclear documentation of medication changes (2) Unfamiliar with patient (2) Patient knowledge of pre-admission medications (1)	Discontinuation not clearly documented (4) Reason for discontinuation not clearly documented(3) Unfamiliar with patient (2) Poor admission notes (1) Misreading medication prescription charts (1) Use of paper charts (1) Inaccurate note taking (1) Medications which were held not being restarted appropriately (1) Time pressure (1)

Are there any other types of error which you think are common on discharge? (n = 5)	Are there any other aspects of discharge prescribing which you think warrant further investigation? (n = 3)
Duration of medication (3) Errors due to patients being discharged on weekends(1) Not documenting benzodiazepines appropriately (1)	Guidance on opiate prescribing at discharge (2) Variability in clinicians (1) Difficulty contacting clinicians (1)

Interns were asked to identify areas of prescribing which warrant further investigation. Guidance on opiate prescribing at discharge was raised as an area of concern.

#### Quantitative survey questions

With regard to 5-point Likert items, most (58.07%) interns agreed or strongly agreed they were familiar with patients they discharge. A majority agreed or strongly agreed (70.96%) that, when they are not familiar with a patient, they could discuss care with someone who is more familiar with a patient. All agreed or strongly agreed that interns who were familiar with patients are less likely to make errors in their discharge prescribing. Most (54.84%) were not comfortable with prescribing for patients whose care they had not been involved in (see Table 3).

**Table 3:** Interns were asked a series of 5-point Likert Style questions relating to experiences writing discharge prescriptions/summaries

Please answer the following questions in relation to your experiences writing discharge prescriptions and discharge summaries in SJH	Strongly disagree (%)	Disagree (%)	Unsure (%)	Agree (%)	Strongly agree (%)
If an intern is familiar with a patient they are less likely to make errors in the patient’s discharge prescription (n = 31)	0	0	0	29.03	70.97
If I am unfamiliar with a patient, I can discuss their care with someone who is more familiar with the patient (n = 31)	3.23	6.45	19.35	58.06	12.9
I am comfortable prescribing for patients whose care I have not been involved in (n = 31)	9.68	45.16	9.68	25.81	9.68
I am familiar with patients I complete discharges on (n = 31)	0	3.23	38.71	54.84	3.23

Interns were asked which sources they used when writing discharge prescriptions, on a scale of 1 to 5, with 5 being always and 1 being never. Results are displayed in Table 4. Inpatient medication lists were always used by all participants, while other sources were less frequently used. This finding may be important when considering the available of medication lists with the EPR implementation.

**Table 4:** Interns were asked to identify the frequency with which four different sources were referenced when preparing discharge prescriptions

Please indicate how often you refer to each of the following when writing discharge prescriptions	Never (%)	Rarely (%)	Sometimes (%)	Often (%)	Always (%)
Patient Kardex (inpatient medication list) (n = 31)	0	0	0	0	100
Clinical Notes (n = 31)	0	9.68	12.9	48.39	29.03
Pre-admission medication reconciliation list (n = 31)	0	6.45	16.13	35.48	41.94
Input from consultants/senior colleagues (n = 31)	0	16.13	45.16	29.03	9.68

## Discussion

### Audit of interventions to improve discharge prescribing

Medications are frequently omitted from discharge prescriptions and discharge summaries in Saint James’s Hospital. Given the substantial body of evidence linking these omissions to adverse drug events and subsequent readmission, as well as inappropriate propagation of inaccurate prescribing in the community, these errors represent a source of preventable iatrogenic risk to patients.

#### Impact of an educational intervention

The educational intervention did not significantly impact discharge prescribing error rates. While there was a trend towards increased communication of medication changes observed in the Post-Intervention cohort, this was not statistically significant.

This lack of impact can be rationalised by the type of educational intervention used. Our educational intervention comprised of didactic teaching session and information bulletins. Both interventions rely on passive dissemination of information, and there is very little evidence to support this as an effective means of improving patient outcomes [24, 29].

#### Impact of EPR

The introduction of EPR significantly reduced discharge prescribing error, with a significant reduction in the primary outcome of proportion of PAM which are prescribed or discontinued with explanation on discharge. This may be explained by EPR design. Prior to introduction of EPR, less than half (41.94%) of interns always referred to admission MR when completing discharge prescriptions, and inpatient medication lists (including current prescriptions) were likely the only or primary source used for discharge prescribing. Following introduction of EPR, the discharge prescribing process integrated patient medication lists with admission MR. This ensures that the discharge prescriber is aware of medications which have been intentionally held, either during admission or on discharge, and this information was not readily available on inpatient medication lists.

The above findings are in line with other studies which examine impact of EPR systems on discharge prescribing error [24, 25]. Direct comparison between studies is often limited by inter-study variability, although a trend towards improved prescribing has been validated in a number of papers [25, 30].

#### Understanding results in the context of EPR design and prescribing habits

In order to rationalise findings, results were contextualised with similar studies, survey data, and discussion with the SJH Informatics Department [24, 25]. Despite improvement in patient safety, a similar study identified a new form of sociotechnical error that was unique to EPR prescribing [25]. Another study noted issues with EPR rigidity as a cause of error [31]. In this study, despite improvement in the primary composite outcome, issues were identified. The rate of deliberate discontinuation of a medication at discharge fell to near zero following introduction following introduction of EPR. Two explanations for this negative finding were identified.

A significant separation of process for discharge prescribing and discharge discontinuation was introduced with EPR. Prior to EPR, paper discharge prescriptions contained an integrated box in which prescribers were prompted to list and rationalise discontinued medications. Issues accessing documentation, and difficulty interpreting documentation were frequently cited by intern prescribers as sources of error in the survey conducted. On SJH EPR, there are now separate tabs for “Discharge Medication Reconciliation” and “Medication Changes and Comments”, and the latter tab is frequently not used when discharging patients. This separation of processes increases number of steps required to list and rationalise discontinued medications.

Access to documentation is important in identifying and rationalising medications which have been discontinued, and the EPR introduction may have reduced this accessibility. When completing the Medications Reconciliation tab, it is not possible to access clinical notes. When completing the Medications Changes and Comments tab, it is not possible to concurrently access any information from the patient’s chart. When asked to identify causes of prescribing an intentionally discontinued medication on discharge, interns frequently cited issues such as “discontinuation not clearly documented”, “poor admission notes” and “reason for discontinuation not clearly documented”. As demonstrated in Table 4, prescribers frequently rely on multiple sources of information when generating discharge prescriptions. Expecting prescribers to navigate in and out of these tabs may detract from the prescribing task at hand and increase the time taken to complete it. Of note, time pressure is frequently listed as a cause of prescribing error in survey data. As some pre-configured electronic patient record systems may not lend themselves to reconfiguration, local awareness of limitations of EPR, education of prescribers about these limitations and trials of work-around solutions have potential to improve patient care. It is essential to ensure such systems are audited appropriately.

Efforts to improve discharge prescribing should focus on making the relevant resources quickly available to the prescriber, in a manner which allows them to identify relevant information. Future quality improvement efforts should attempt to integrate clinical notes and input from senior colleagues (in addition to admission MR) in the discharge prescribing process. Providing teaching to non-consultant hospital doctors on this topic is unlikely to be adequate in developing a proactive service. The role of clinical pharmacists in medication safety is essential in all health systems, irrespective of e-health infrastructure. In the survey of intern doctors, they reported that medication omissions on admission were the most likely cause of omissions at discharge. This highlights the importance a pharmacist led MR on admission. Pharmacist-led medication reconciliation is effective at reducing discharge prescribing errors in health systems electronic patient records are instituted [32]. This is a cost-effective method of reducing error, and clinicians should advocate for discharge MR implementation where possible [17].

### Strengths and limitations

Strengths of this study include assessment of two interventions in the same institution. The study is unique in examining interventions in terms of changes to hospital-wide clinical practice in a pragmatic setting, rather than surrogate markers like clinician performance in a standardised assessment. It assesses two interventions in the same institution, and includes an educational intervention which is passive in nature, reflecting the most widely available mechanism for addressing discharge prescribing issues.

Limitations of this study include variation between study groups. There was a relative reduction in number of PAM per patient in the Post-EPR study group, compared to the Pre-Intervention and Post-Education study groups. Given that number of PAM did not correlate significantly with primary or secondary outcomes, this did not likely alter our interpretation of the study results. Further, this study was conducted on a sample of 1,218 PAM (162 discharged patients) in one hospital system. Results may not be directly applicable to other hospital systems.

## Conclusion

There is a significant body of evidence in the Irish context that the omission of medications from discharge prescriptions and discharge summaries has an impact on patient care, and is therefore a strong marker of iatrogenic risk [2, 3, 5–8, 10–12]. This study found that a multi-component educational intervention had a small effect on improving prescribing errors and communication at discharge. However, the implementation of an electronic patient record with a discharge prescribing function significantly reduced prescribing errors and omissions at discharge.

## Supplementary Information

**Additional file 1**. Survey tool developed to obtain information on prescribing habits of junior doctors when preparing discharge documentation.

## Data Availability

The datasets analysed during the current study are available in an irrevocably anonymised format from the corresponding author on reasonable request.

## Abbreviations

PAM: Pre-admission medications prescribed prior to hospitalization
EPR: Electronic patient record
EMR: Electronic medical record
GP: General practitioner
MR: Medicine reconciliation
SJH: Saint James’s Hospital
PCPD: Proportion correctly prescribed on discharge
PIPD: Proportion incorrectly prescribed on discharge
