# Supplementary material for: Assessment of an electronic patient record system on discharge prescribing errors in a Tertiary University Hospital
Source: BMC Med Inform Decis Mak. 2021 Jun 21;21:195. doi: 10.1186/s12911-021-01551-5 (PMC8218465; doi:10.1186/s12911-021-01551-5)
Supplement: Supplementary file 1 — Additional file 1. Survey tool developed to obtain information on prescribing habits of junior doctors when preparing discharge documentation. [file 12911_2021_1551_MOESM1_ESM.pdf]

Assessment of an Electronic Patient Record System on Discharge Prescribing Errors in a Tertiary University Hospital

Additional Material: Survey on Discharge Prescribing for Interns in SJH

Authors:

Michael O'Shea[1,2,3], Cormac Kennedy[1,3], Eileen Relihan[1], Kieran Harkin[4], Martina Hennessy[1,3]and Michael Barry[1,3]

1 – Saint James's Hospital, Dublin, Ireland; 2- Dublin Southeast Network Academic Track Internship, Dublin, Ireland; 3 – Trinity College Dublin, Ireland; 4 – Inchicore Family Doctors, Dublin, Ireland

Corresponding author: Michael O'Shea

Email: osheam12@tcd.ie

Tel: +1 602 348 3534

Address: 55 O'Reilly Avenue, Ceannt Fort, Kilmainham, Dublin 8, D08PR2A, Ireland.

File name: Survey on Discharge Prescribing for Interns in SJH

File Format: PDF

Title of Data: Survey of Interns on Discharge Prescribing in SJH

Description of Data: This document contains a survey of first year junior doctors (interns), which was conducted on the 9th of January 2018. This survey was first piloted for acceptability and ease of use on final year medical students.

Survey on Discharge Prescribing for Interns in SJH

This is a survey which is a component of a quality improvement audit in Saint James's Hospital. By completing this survey, you consent to have your answers included to this project, and any subsequent publications or presentations that may result from the project. Please do not include any information which could identify you or anyone else.

How long have you been an intern? ____ months and ____weeks
How long have you been an intern at SJH? ____ months and ____weeks
What medical school did you attend? _______________
What type of medical program (eg postgraduate, undergraduate) did you complete? __________

Prescribing at Discharge

Please answer the following questions in relation to your experiences completing discharge prescriptions and discharge summaries for inpatients in SJH.

Please indicate to what extent you agree/disagree with the following statements	Strongly disagree	Disagree	Unsure	Agree	Strongly Agree
I am satisfied with my ability to complete discharge summaries and prescriptions in SJH					
If an intern is familiar with a patient they are less likely to make errors in the patient's discharge prescription					
It is important to indicate which medications are discontinued on discharge prescription					
I have received adequate training on discharge prescribing					
If I am unfamiliar with a patient, I can discuss their care with someone who is more familiar with the patient					
I would like further teaching in pharmacology and practical prescribing as part of my internship year					
A prescribing safety exam at the end of intern year would improve patient care					
I am comfortable with prescribing for patients whose care I have not been involved with					

How often do you think the following occurs?	Never	Not often	Sometimes	Often	Always
If there has been a change in a patient's medication, I attempt to find the reason for this change in their records before completing the discharge prescription and summary					
If I have a question about a patient's medication on discharge, I can talk to someone more senior about the issue					
If I am unfamiliar with a patient, I can discuss their care with someone who is more familiar with the patient					
I am familiar with patients I complete discharges on					

Factors which contribute to discharge prescriptions

Please indicate how often you refer to each of the following items when writing a discharge prescription.

	Never	Rarely	Sometimes	Often	Always
Patient Kardex					
Clinical notes					
Pre-admission medicine reconciliation list					
Input from consultants/ senior colleagues					

Please turn over

How frequently do you think you make the following errors when writing discharge prescriptions?	> Once a day	Once a day	> Once a week	Once a week	> Once a month	Once a month	Never
Omission of a medication							
Error relating to dose, frequency or formulation							
Prescription at discharge of a medication which was intentionally discontinued during hospital stay							
Communication error							

For each of the errors mentioned above, please indicate why you think they occur in discharge prescribing?

1. Omission of medication:

2. Error relating to frequency, dose or formulary:

3. Communication error:

4. Prescription of a medication that was intentionally stopped during inpatient stay:

Are there any other types of errors which you think are common on discharge?

Yes No

If yes, please elaborate:

<u>Possible changes to prescribing</u>

Please indicate which changes you think will be successful in reducing medication errors, by grading each of the options on a scale of 1-5, where 1 is no reduction and 5 is substantial reduction.

Change	1	2	3	4	5
Further education of Non-Consultant Hospital Doctors					
Pharmacist performing medicine reconciliation at discharge					
Introduction of electronic prescribing					
Provision of a patient-held 'current medicines list card' to all patients, which is updated at discharge					
Provision of a patient-held memory key or account on a secure server containing a medication list which is updated at discharge					
Discharge prescriptions indicate if they are supplemental primary care prescription, or if they are to replace previous primary care prescriptions					
Including prescriber contact details (eg a healthmail address) in discharge prescriptions or discharge summary					

Please indicate if there are any changes you think would reduce prescribing error at discharge, which are not included the list above. Please elaborate on these changes:

Are there any other aspects of discharge prescribing which you think warrant further investigation?

Do you think it would be helpful to have a shared primary care/secondary care regional formulary for common medicines?

Yes No

If yes, please elaborate:
